# Thrombosis in Neuromuscular Medicine: Current Evidence, Unmet Needs, and Future Directions

**DOI:** 10.3390/jcm15082810

**Published:** 2026-04-08

**Authors:** Zhi Xuan Quak, Furene Wang, Stacey K. H. Tay, Pei Lin Koh, Eng Soo Yap, Kay Wei Ping Ng

**Affiliations:** 1 Division of Neurology, Department of Medicine, National University Hospital, Singapore 119074, Singapore; kay_wp_ng@nuhs.edu.sg; 2 Division of Paediatric Neurology, Department of Paediatrics, Khoo Teck Puat—National University Children’s Medical Institute, National University Hospital, Singapore 119074, Singapore; furene_wang@nuhs.edu.sg (F.W.); stacey_tay@nuhs.edu.sg (S.K.H.T.); 3 Department of Paediatrics, Yong Loo Lin School of Medicine, National University of Singapore, Singapore 119228, Singapore; 4 Division of Paediatric Haematology and Oncology, Department of Paediatrics, Khoo Teck Puat—National University Children’s Medical Institute, National University Hospital, Singapore 119074, Singapore; pei_lin_koh@nuhs.edu.sg; 5 Division of Haematology, Department of Haematology-Oncology, National University Cancer Institute, Singapore 119074, Singapore; eng_soo_yap@nuhs.edu.sg; 6 Department of Laboratory Medicine, National University Hospital, Singapore 119074, Singapore

**Keywords:** neuromuscular disease, thrombosis, stroke, embolism

## Abstract

Venous thromboembolism (VTE), comprising deep vein thrombosis and pulmonary embolism, is an important but under-recognised complication in neuromuscular diseases. In adults, emerging epidemiological data suggests increased VTE occurrence in conditions such as Amyotrophic Lateral Sclerosis, myotonic dystrophy, myasthenia gravis, inflammatory neuropathies, inflammatory myopathies, and POEMS syndrome. This heightened risk reflects not only disease-related immobility but also disorder-specific biological mechanisms, including inflammation, endothelial dysfunction and cardiomyopathy-related stasis. Therapies such as corticosteroids, IVIG-related hyperviscosity, long-term central venous access, perioperative immobility, critical illness, and complex orthopaedic procedures have prothrombotic effects. Despite this multifactorial risk profile, disease-specific guidance for thromboprophylaxis is lacking, and current practice relies heavily on extrapolation from general medical and surgical recommendations rather than data derived from neuromuscular cohorts. In children and adolescents, the VTE burden is less well-characterised, but events have been reported in Duchenne and Becker muscular dystrophy, congenital myopathies, and spinal muscular atrophy particularly with advanced motor impairment, severe cardiomyopathy, ventilatory insufficiency, and prolonged hospitalisation. Beyond venous events, selected neuromuscular disorders also exhibit increased arterial thrombosis risk. Myotonic dystrophy and dystrophinopathies are associated with cardiomyopathy and arrhythmia that predispose to systemic embolism and stroke, while inflammatory myopathies may demonstrate arterial events related to vasculitic or endothelial processes, although overall evidence remains limited. This review summarises available empirical and epidemiological evidence on venous and arterial thrombosis across adult and paediatric neuromuscular disorders, outlines disease-specific mechanistic pathways, examines treatment-related contributors, and highlights key evidence gaps that must be addressed to guide rational and targeted prophylaxis strategies in this complex, heterogeneous population.

## 1. Introduction

Venous thromboembolism (VTE), comprising deep vein thrombosis and pulmonary embolism, is an important but under-recognised complication in neuromuscular diseases. Patients with neuromuscular diseases experience greater immobility, putting them at risk of VTE. In addition, disease-specific mechanisms including inflammation, endothelial dysfunction and cardiomyopathy-related stasis contribute to an increased risk of thrombosis in certain neuromuscular conditions [1]. Epidemiological data suggests a wide range of differing risks of VTE among neuromuscular diseases, which likely reflects the variation in biological risk profiles between different neuromuscular diseases [2,3].

Arterial thrombosis is also manifested at an increased rate in certain neuromuscular disorders, predisposing to systemic embolism and stroke.

Beyond the inherent thrombotic risk due to the underlying neuromuscular disease, patients with neuromuscular diseases may also receive therapies that may predispose them to thrombosis, such as glucocorticoids and intravenous immunoglobulin (IVIG) with its related hyperviscosity [4,5]. Other associated procedures such as long-term central venous access and a need for complex orthopaedic procedures with higher risks of perioperative immobility have prothrombotic effects [3]. These patients may also have an increased risk of critical illness that may put them at risk of thrombosis.

Despite this multifactorial risk profile, disease-specific guidance for thromboprophylaxis is lacking, and current practice relies heavily on extrapolation from general medical and surgical recommendations rather than data derived from neuromuscular cohorts. In children and adolescents, the VTE burden is less well-characterised, but events have been reported in a spectrum of hereditary neuromuscular conditions, particularly in patients with advanced motor impairment, severe cardiomyopathy, ventilatory insufficiency, and prolonged hospitalisation [6].

This review summarises available empirical and epidemiological evidence (Table 1) on venous and arterial thrombosis across different adult and paediatric neuromuscular disorders, outlines disease-specific mechanistic pathways, examines treatment-related contributors, and highlights key evidence gaps that must be addressed to guide rational and targeted prophylaxis strategies in this complex, heterogeneous population.

## 2. Motor Neuron Diseases

Motor neuron diseases are a heterogeneous group of progressive neurodegenerative disorders characterised by the selective loss of upper and/or lower motor neurons, leading to muscle weakness, atrophy, and eventual paralysis. Among the more prevalent and clinically significant forms are Amyotrophic Lateral Sclerosis (ALS), a typically adult-onset disorder marked by combined upper and lower motor neuron degeneration [24], and spinal muscular atrophy (SMA), a primarily early-onset genetic condition caused by homozygous SMN1 deletion [25].

### 2.1. Amyotrophic Lateral Sclerosis

Clinical features of ALS include progressive loss of muscle bulk and strength in the limbs, swallowing and speech impairments and respiratory compromise. A combination of lower and upper motor neuron features is seen, usually asymmetric, with atrophy and fasciculations co-existing with spasticity and hyperreflexia. There is an absence of sensory deficits. The natural course of the disease is of progressive disability eventually leading to respiratory and nutritional failure. Death commonly occurs within 2–5 years of disease onset [24]. While death is most commonly due to respiratory failure, VTE has also been reported as a cause of sudden death [26].

A recent meta-analysis on the incidence of VTE in patients with ALS [7] showed that the pooled annual incidence of VTE across the eight included studies involving 26,758 patients was 22 cases per 1000 patient-years (95% CI, 18–27). Included studies were of variable quality and incidence rates ranging from 19 to 111 cases per 1000 patient-years, although incidence rates converge in studies with larger sample sizes. Compared with estimates of the baseline risk of VTE in a general population of about 1–2 per 1000 person-years [2], the incidence of VTE is clearly increased in ALS. Risk factors associated with VTE in patients with ALS include a prior VTE history, being on non-invasive ventilation, immobility and a worse functional status [7]. This supports the hypothesis that the mechanism of VTE in ALS is contributed to by immobility and venous stasis. However, other mechanisms of systemic inflammation [27] and erythrocyte dysfunction [28] may also have a role.

The largest and most recent of the included studies in the meta-analysis is a 2024 UK hospital record linkage study, retrospectively evaluating the records of 21,163 patients [8]. The incidence of VTE in the ALS cohort was 18.8 per 1000 patient-years, with a higher relative risk of VTE in ALS than controls (adjusted HR 2.7, 95% CI 2.4 to 3.0), with ALS patients who were less than 65 years old having a five-fold higher relative risk of VTE. Overall, however, the higher risk of developing VTE in ALS patients was not higher than that reported in other chronic neurological conditions associated with immobility, such as multiple sclerosis [8].

Considering that conditions with comparable VTE risk to ALS, such as multiple sclerosis and cancer, do not have routine recommendations for VTE prophylaxis, and the lack of clarity over the risk-benefit ratio, it may be extrapolated that primary VTE prophylaxis in ALS is likely best avoided. In addition, risk of falls in ALS patients is increased due to their disability, adding to the increased possibility of harm from empiric anticoagulation. In light of this, less aggressive prophylaxis methods can be considered, such as aspirin, graduated compression stockings, and intermittent pneumatic compression. However, the authors acknowledged some of the limitations on evidence-based efficacy and the additional difficulty in patients who may still retain limited mobility. A suggested strategy would be to initiate pharmacological prophylaxis only in chairbound patients who are at lower risk of falls [8].

Current published guidelines [29,30,31] are generally structured around a multidisciplinary framework that addresses diagnosis, disease modification, symptom management and end-of-life care. An increase in thrombotic risk, particularly VTE, and need for vigilance is acknowledged in the guidelines [29,30,31], although recommendations for prophylactic anticoagulation are not made due to lack of evidence. Non-pharmacological interventions such as compression stockings, limb elevation and passive physiotherapy are recommended.

The increase in VTE risk in ALS patients underscores the importance of maintaining heightened vigilance, especially in patients with advanced ALS who present with limb swelling, pain, or breathlessness. While such symptoms may be indicative of musculoskeletal pain related to immobility or progressive respiratory muscle weakness, an evaluation for VTE should be considered when clinical suspicion arises.

The risk of arterial events like stroke and myocardial infarction is less well-recognised in ALS and are not discussed in guidelines [29,30,31]. However, there is emerging evidence that incidence of arterial events is also increased. Recent cohort studies from South Korea using a national database showed that, compared with a control group, ALS was associated with a higher incidence of ischaemic stroke (7.8 vs. 3.2 per 1000 person-years) [9] and myocardial infarction (26.2 vs. 3.2 per 1000 person-years) [10]. In total, 659 ALS patients, age and sex matched to 10,927 controls, were included in the analysis. Administrative data was used in the identification of cases and determination of outcomes, which has inherent risks of misclassification and information bias. Additionally, the studies assessed disability using generic national disability registration system instead of the widely accepted ALS Functional Rating Scale (ALS-FRS), limiting the interpretation of the conclusions the authors draw regarding the impact of physical disability on thrombosis risk.

Nonetheless, the studies found that stroke risk did not significantly differ between disabled and non-disabled ALS patients, which the authors suggested that, unlike for VTE, immobility and physical disability did not explain the association between ALS and stroke. Furthermore, traditional vascular risk factors (like increased Body Mass Index) were less prevalent in ALS patients [32], suggesting that other mechanisms like hypercoagulability due to systemic inflammation and endothelial dysfunction may be the primary drivers of the thrombotic risk [27].

Further studies to corroborate the observations made in the South Korean cohort are required before definitive conclusions or actionable recommendations can be made regarding arterial thrombosis in ALS.

### 2.2. Spinal Muscular Atrophy

Spinal muscular atrophy (SMA) is an autosomal recessive neuromuscular disorder caused by homozygous deletion of the survival motor neuron 1 gene (SMN1). The incidence of SMA is approximately 1 in 10,000 live births with a carrier frequency of approximately 1 in 50 people [25]. SMA is divided into four clinical subtypes which vary in age of onset, highest motor milestone achieved, and prognosis. They range from type I, with infantile onset, no independent sitting and poor early survival, to type IV, with adult-onset, milder weakness with preserved ambulation and normal life expectancy. Types II and III have intermediate onset and milestone achievement (sitting in type II and walking in type III), and progressively better prognosis [33].

Although SMA is typically characterised by progressive motor neuron loss leading to skeletal muscle weakness and denervation-induced atrophy [33], various studies and case reports suggest that the vascular endothelium is vulnerable to diminished SMN expression [34,35,36]. This has manifested in patients with severe SMA presenting with distal digital necrosis, with vascular histology showing thrombotic occlusion of the small vessels [35,36]. Autopsies of SMA infants with severe SMA show decreased von Willebrand factor (VWF) in the spinal cord endothelium, which is a widely used cytosolic marker of endothelial cells and endothelial cell health, suggesting that compromised endothelial integrity may contribute to thrombosis [34,37]. Additionally, autonomic dysfunction in severe SMA also likely contributes to the observed vascular perfusion abnormalities [35,36,38].

The incidence of venous thrombotic events has not been systematically studied in SMA and is likely rare. Factors that may predispose to VTE include venous stasis due to immobility, a hypercoagulable state due to endothelial dysfunction, and transitory risk factors like acute hospitalisations and surgical procedures. A case report of an adolescent patient with SMA type III suffering an intraoperative VTE during surgery for contracture release illustrates the presence of multiple predisposing risk factors in SMA patients [39].

While arterial thrombotic risk for SMA patients has also not been systematically studied, data from adult and paediatric patients suggest an altered lipid and glucose metabolism profile [40,41]. This may predispose them to long-term cardiovascular risk, and authors recommend regular metabolic screening and appropriate nutritive support [40,41].

Congenital heart defects have also been reported in infants with severe SMA type I, potentially due to early endothelial dysfunction, as vascular endothelial precursors are essential for heart development [42]. Structural abnormalities of the heart most frequently involved the atrial and ventricular septum and the cardiac outflow tract [42]. In contrast, cardiac conduction defects affect SMA patients across the spectrum of severity and include atrial fibrillation and flutter [42]. The finding of structural cardiac abnormalities or rhythm disturbances that increase stroke risk prompts consideration of anticoagulant therapy.

## 3. Muscular Dystrophies

Muscular dystrophies are a clinically heterogeneous group of inherited muscular disorders causing progressive muscular weakness and atrophy. They arise from genetic mutations in genes coding for proteins involved in aspects of muscle structure and function [43]. Beyond involvement of skeletal muscles, many dystrophies have cardiac and other systemic manifestations. Timing of symptom onset can range from birth to adult life and inheritance patterns vary.

### 3.1. Dystrophinopathies

Dystrophinopathies [Duchenne’s (DMD) and Becker’s (BMD) muscular dystrophy] are X-linked muscular dystrophies caused by mutations in the dystrophin gene, and primarily affect boys from childhood, but can also have manifestations in female carriers. They are among the most common muscular dystrophies in childhood and adolescence, with a prevalence of about 7 per 100,000 males [44]. Patients experience progressive weakness, starting at the trunk and progressing to the limbs. Cardiomyopathy and respiratory compromise from scoliosis and respiratory muscle weakness often limit survival.

Beyond the motor and cardiopulmonary impairment, patients can experience thrombotic events such as VTE, strokes and myocardial infarction [6] due to a combination of chronic inflammation, cardiac dysfunction, and treatment-related thrombosis. Glucocorticoids are frequently used for delaying muscle degeneration and preserving cardiac function but are well-known to be thrombogenic [5]. It had also been previously suggested that patients with DMD may have a hypercoagulable state with abnormalities in fibrinolysis, that may be associated with cardiac dysfunction [45].

Orthopaedic procedures are also commonly undertaken for fractures, scoliosis and contractures, and have an associated risk of thrombosis. Long bone and spine fractures occur at an increased incidence rate in children and adults with DMD, with up to 25% of boys experiencing long bone fractures [46]. Their occurrence relates to decreased bone density, further exacerbated by the chronic use of glucocorticoids in the treatment for DMD [46,47]. Patients recovering from fractures experience increased immobility and are exposed to a post-traumatic hypercoagulable state. Endothelial damage caused by the fractured ends of long bones further contributes to the thrombotic mechanisms [48]. General surgical guidelines [49] recommend pharmacological prophylaxis in long bone fractures perioperatively for VTE but have no specific recommendations for patients with dystrophinopathies. Beyond the thrombotic causes of pulmonary embolism, fat embolism also needs to be considered, particularly in the setting of acute alteration in consciousness, respiratory distress and a typical skin rash [50].

Elective procedures like tendon release and spinal fusion for scoliosis are frequently undertaken in patients with DMD to improve mobility and preserve lung function [51]. While the risk of VTE is recognised in these procedures in a general population, and pharmacological prophylaxis is generally recommended [52], it cannot be easily extrapolated to patients with DMD, as abnormalities in haemostasis in these patients may tilt the balance [53].

Highlighting this complexity and uncertainty, the results of a patient registry-based survey involving 351 DMD patients did not indicate a DMD-specific risk for thromboembolic events beyond the risk for comorbidities of chronic immobility and cardiac insufficiency, with a history of thromboembolic events found in 0.6% of DMD patients surveyed. These results even suggested a mild bleeding risk with 21% of participants reporting a bleeding tendency [54]. While the survey is limited by its study of patient-reported outcomes without more objective markers, the observations made are nonetheless valuable to direct further studies to more robustly evaluate bleeding and thrombotic risks in this population.

Regardless, there are anecdotal reports [55] of spontaneous DVT after spinal fusion, hence clinical vigilance for peri-procedure VTE should not be overlooked. Overall, decisions for VTE prophylaxis require individualisation of VTE and bleeding risk, with consideration given to the patient’s level of immobility, especially in the settings of surgery or hospitalizations. Validated tools to guide these decisions are currently lacking and can be a consideration for further study.

Cardiac complications including dilated cardiomyopathy, arrhythmias and congestive cardiac failure invariably occur in the later stages of DMD and BMD [56]. Onset of cardiac disease occurs in the teenage years, and limits life expectancy. Management initially focuses on the prevention of cardiac remodelling and subsequently shifts towards symptomatic palliation in later stages [56]. Coagulation pathways are activated in patients with severe left ventricular dysfunction [45], and patients are predisposed to complications of cardioembolism, such as ischaemic strokes [6,57]. The risk of embolic stroke appears to be correlated primarily with dilated cardiomyopathy and low ejection fraction, and less dependent on the presence of atrial fibrillation or a detected intracardiac thrombus [58]. However, routine anticoagulation in these patients is not recommended in the guidelines [51,56] beyond the setting of an acute hospitalisation or perioperatively. The detection of a cardiac thrombus or atrial fibrillation would prompt the initiation of anticoagulation, based on established risk criteria [59].

Patients with severe cardiomyopathy may choose to undergo implantation of a left ventricular assist device (LVAD), either as a bridge to cardiac transplant or as destination therapy [51,56]. As pump thrombosis is a serious complication of LVAD therapy, lifelong anticoagulation (usually with a vitamin K antagonist) and antiplatelet therapy are usually recommended [60].

### 3.2. Myotonic Dystrophy and Other Congenital Muscle Diseases

Apart from dystrophinopathies, other muscular dystrophies have features that warrant the consideration of thrombotic risk. Individually, they are rare diseases, and hence disease-specific incidences of thrombotic events are hard to quantify, with no available disease-specific recommendations for thromboprophylaxis.

A review of atrial fibrillation/flutter (AF/AFL) in myopathies estimated the prevalence of AF/AFL in primary myopathies to be around 6.1% [61]. Muscular dystrophies with significant associations with cardiac arrhythmia: myotonic dystrophy type 1 and type 2 (DM1 and DM2), emery-dreifuss muscular dystrophy (EDMD), and limb-girdle muscular dystrophies 1B (LGMD1B) caused by lamin A/C (LMNA) mutations have been associated with embolic strokes in the setting of atrial fibrillation [62,63,64]. Consequently, The American Heart Association statement [65] recommends thromboprophylaxis in children with neuromuscular disease, normal ventricular function and atrial fibrillation or flutter.

Beyond anticoagulation in the settings of atrial fibrillation and flutter, there is lack of evidence for routine stroke prophylaxis with other agents like aspirin [66]. Fall risk related to underlying neuromuscular weakness, postural hypotension and syncope associated with autonomic dysfunction in DM1 [67] need to be considered when deciding on anticoagulation [66].

With regard to the risk of venous thromboembolism, retrospective data suggests an increased risk of VTE in patients with myotonic dystrophy compared with other muscular dystrophies. Sochala et al. reviewed the data of 1148 patients with myotonic dystrophy and 1662 patients with other inherited myopathies (facioscapulohumeral, dystrophinopathy, mitochondrial, glycogen- and lipid-storage diseases, limb-girdle muscular dystrophies, nucleopathies, collagen VI-related disorders, myofibrillar, and congenital) [11]. They found an incidence of 8.3 per 1000 patient-year (95% CI, 6.6–10.3) and 10.3% cumulative incidence (95% CI, 7.8–12.7) of ≥1 VTE event occurring in patients of myotonic dystrophy. The same study estimated the incidence of VTE in the other myopathies at 1.6 per 1000 patient-year incidence (95% CI, 1.0–2.5) and 2.2% cumulative incidence (95% CI, 1.1–3.3). VTE was unprovoked in 58.5% of patients with myotonic dystrophy, and in 30% of the other myopathies. Pulmonary embolism was also the second most frequent cause of cardiovascular death (31.3%) after sudden cardiac death.

In their multivariable analysis, among the 2810 patients with inherited myopathies, predictors of VTE were myotonic dystrophy (HR, 5.35; 95% CI, 2.85–10.0; *p* < 0.0001), increasing age (HR, 1.02; 95% CI, 1.00–1.03; *p* = 0.031), personal history of VTE (HR, 4.56; 95% CI, 2.28–9.10; *p* = 0.0001), obesity (HR, 2.66; 95% CI, 1.64–4.32; *p* < 0.0001), high Walton score (HR, 1.20; 95% CI, 1.09–1.32; *p* = 0.0003), cancer (HR, 4.32; 95% CI, 2.05–9.08; *p* < 0.0001), conduction disease (HR, 1.63; 95% CI, 1.06–2.51; *p* = 0.025), and ambulation loss in patients with myotonic dystrophy (HR, 7.18; 95% CI, 1.88–27.4; *p* = 0.004). The authors hypothesised that this association between VTE and myotonic dystrophy may involve the toxic effect of RNA, resulting in the missplicing of factors involved in the coagulation process [11].

To address the above, it has been suggested that the decision to commence long-term anticoagulation in these patients also needs to be balanced against their risk of bleeding complications given their fall risk and possible treatment non-compliance given variable intellectual impairment. Women should be counselled on the risks of oral contraceptives and post-menopausal oestrogen replacement therapy given the VTE risk [68].

Overall, the Sochala study, by comparing a large population of myotonic dystrophy patients to other inherited myopathies and a community-based population, convincingly demonstrated that the VTE risk in myotonic dystrophy patients was specific to myotonic dystrophy rather than muscular dystrophy in general.

In another study, LMNA mutations (related to emery-dreifuss muscular dystrophy) appear to be independently associated with venous and arterial thrombotic risk even after adjustment for possible confounders, including atrial tachyarrhythmias and left ventricular ejection fraction that occur due to concomitant cardiomyopathy. LMNA mutation carriers were found to have altered platelet function and increased thrombin generation, leading to a prothrombotic phenotype [69].

At present, there are no generalisable recommendations for VTE prophylaxis in these subgroups. Physicians treating these conditions should be aware of the potential increased risk of VTE, and exercise appropriate clinical vigilance in applying existing VTE guidelines. It is suggested that a more systematic VTE prophylaxis, especially for myotonic dystrophy, should also be extended to any medical or surgical setting [11].

## 4. Acquired Inflammatory/Immune-Mediated Neuromuscular Diseases

Acquired inflammatory or immune-mediated neuromuscular diseases are an important cause of weakness and disability across all age groups, though more commonly seen in adults. They can present subacutely or have a more chronic relapsing course. As autoimmune diseases, they may possess innate hypercoagulability tendencies as systemic inflammation suppresses fibrinolysis and shifts the balance towards procoagulant factors [70]. These diseases include myasthenia gravis (MG), Guillain Barre syndrome (GBS), chronic inflammatory demyelinating neuropathy (CIDP) and the spectrum of inflammatory myopathies.

Despite having varying pathologies and clinical trajectories, they share overlapping features of progressive or fluctuating weakness of the limbs, bulbar and respiratory muscles, causing significant functional impairment. In severe manifestations, they may be life threatening, necessitating ventilatory support in a critical care setting. Their treatment often involves immunomodulatory therapy, including glucocorticoids, IVIG and plasmapheresis. The thrombotic risk in these conditions stems from the above commonalities of disease-related immobility, systemic inflammation, critical illness and treatment-related complications. These risks may be especially significant when these patients deteriorate sufficiently to require ICU care, with the more common neuromuscular disorders seen in ICU being MG and GBS [71].

The 2016 Neurocritical Care Society guidelines noted that critically ill patients with neuromuscular diseases (e.g., GBS, MG) are at high risk for VTE, but no studies have directly evaluated VTE prophylaxis in these specific populations. Therefore, recommendations were extrapolated from evidence in hospitalised/critically ill medical patients and patients with spinal cord injury. Based on that indirect evidence, the panel recommends pharmacologic prophylaxis with prophylactic-dose unfractionated heparin, low-molecular-weight heparin, or fondaparinux as first-line therapy, with intermittent pneumatic compression (IPC) used when bleeding risk precludes anticoagulation. They suggest combining pharmacologic plus IPC prophylaxis when feasible, reserving graduated compression stockings (GCS) only when neither anticoagulation nor IPC can be used, and continuing prophylaxis for at least the duration of acute hospitalisation or until ambulation returns. The strength of evidence ranged from moderate (for pharmacologic prophylaxis and IPC use) to low or very low (for combination therapy, GCS use, and duration), reflecting the absence of direct trials in neuromuscular ICU patients and reliance on extrapolated data [72].

### 4.1. Myasthenia Gravis (MG)

In myasthenia gravis, pathogenic antibodies attack the neuromuscular junction, causing an interruption in neuromuscular transmission resulting in clinical manifestations of fluctuating, fatigable weakness. Most cases are positive for antibodies to the acetylcholine receptor, and less commonly muscle specific kinase or low-density lipoprotein-related peptide 4. Estimations of incidence of VTE in MG come from large population datasets. A US retrospective study utilising insurance claims dataset estimated 6.5% of patients with MG prescribed IVIG are diagnosed with VTE [73]. A British study utilising various hospital admission databases estimated rate ratios of VTE in an English dataset of 2.26 (95% CI, 2.00–2.54) for patients with MG versus a comparison cohort [12]. A Swedish study of autoimmune diseases and thrombosis showed an incidence rate of pulmonary embolism in MG of 7.21 (95% CI, 4.86–10.31) in the first year after diagnosis [74].

Thrombosis in MG may be related to the underlying inflammatory state [75]. However factors related to immobility, critical care or rescue therapies likely have a greater contribution, as suggested by a US study utilising admission data on 5502 MG patients, which showed that DVT occurrence was significantly more common, albeit still low at 0.39% in patients in myasthenic crisis, compared with no DVT occurrence in patients not in crisis [76]. In comparison, the pooled prevalence of VTE in a meta-analysis of 27,344 patients in intensive care units was 10.0% [77].

Thymic pathology is present in up to 80% in seropositive MG [78], and thymectomies are performed in MG patients to remove thymic tumours and for the control of MG symptoms. Patients with thymic cancers are at an increased risk of thrombosis; however, this is somewhat less than with other solid organ tumours like gastrointestinal, ovarian, brain and lung malignancies [79]. A Chinese single-centre study on patients undergoing thymectomy showed a VTE incidence of 4.6% in those with benign thymic disease and 14.5% in those with malignant disease [80]. Upper limb DVT from central venous thrombosis can also occur because of direct tumour compression [81], albeit a rare complication. At present, pharmacologic thromboprophylaxis is still best reserved for critically ill patients in intensive care units, as per 2016 Neurocritical Care Society guidelines [72].

### 4.2. Guillain Barre Syndrome (GBS)

GBS is an inflammatory polyradiculoneuropathy that typically presents with acute flaccid paralysis. In severe cases, it can progress to bulbar, respiratory and autonomic involvement. Antecedent infection is frequently reported, reflecting an aberrant immune response with a cross-reaction of antibodies with peripheral nerve components. In GBS, VTE risk is associated with immobility, underlying systemic inflammation and the use of IVIG, especially in the intensive care setting [82].

Although commonly recognised as a frequent complication of GBS, there is a lack of systematic review on the incidence of VTE in GBS. In a retrospective database analysis of U.S. National Inpatient Sample (NIS) data, out of 40,670 patients who received IVIG, there were 375 cases of VTE/PE in 13,115 patients with GBS who were treated with IVIG, with an odds ratio of 1.36 (95% CI, 1.12–1.66) [13]. In a single-centre English retrospective study on 73 GBS inpatients, despite prophylactic anticoagulation being used in 50 patients who were admitted with immobility, 6% (*n* = 3) of these still developed symptomatic DVT, mostly occurring in the first few months after onset [83].

Current guidelines highlight the need for vigilance regarding VTE and recommend VTE prophylaxis particularly in the setting of immobility [84,85]. There is robust evidence for pharmacologic and mechanical VTE prophylaxis in ICU populations [82], however it is not specific for GBS or neuromuscular patients. Bleeding risk with anticoagulation cannot be ignored, as highlighted by a recent case series of 41 patients with GBS receiving pharmacological prophylaxis, where no VTE events occurred but 24.4% of patients experienced haemorrhagic events, in particular tracheostomy bleeds [86].

In summary, although VTE is recognised as a complication in GBS, the incidence is poorly defined and current prophylaxis strategies are extrapolated from larger populations. This highlights the need for disease-specific evidence to balance bleeding and thrombotic risk. Above evidence, however, is still consistent with 2016 NCS guidelines [72] highlighting the need for VTE prophylaxis in immobile and critically ill GBS patients, particularly if they receive IVIG, but with continued vigilance for haemorrhagic events.

### 4.3. Chronic Inflammatory Demyelinating Polyneuropathy (CIDP) and Autoimmune Nodopathies (AN)

CIDP is an immune disorder of the peripheral nervous system, characterised by progressive or relapsing motor or sensory dysfunction and demyelinating features on electrodiagnostic testing. Clinically, it has a wide spectrum of disease manifestations, severity and disability.

Data on the incidence of VTE in CIDP is sparse, and CIDP is notably absent from reviews of VTE associated with immune diseases [70,74]. Likewise, CIDP guidelines do not make specific reference to VTE risk or prophylaxis recommendations [87].

IVIG is frequently used as first-line treatment for CIDP, and most reports and studies of thrombotic events in CIDP patients relate to IVIG use. A single-centre retrospective study done in the U.K., comprising mostly of patients with CIDP (*n* = 61) or Multifocal Motor Neuropathy (*n* = 41) who are on IVIG, showed an incidence rate of arterial and venous events of 35.7 (95% CI, 18.2–68.9) and 22.3 (95% CI, 9.6–51.2) per 1000 patient-years, respectively [14]. Interestingly, none of the events occurred with the first IVIG infusion, and the event group had a higher rate of vascular risk factors.

Another earlier retrospective single-centre study on 62 patients with inflammatory neuropathy also showed that previous coronary disease and immobility at time of treatment with IVIG independently predicted thromboembolic events [88]. This suggests that in CIDP, thrombotic risk, especially for arterial events, may be increased by concurrent IVIG use especially in patients with predisposing vascular risk factors and immobility. The same study also found that administration of daily doses ≥ 35 g of IVIG correlated significantly with occurrence of early thromboembolic complications [88]. It is unknown that, independent of IVIG use and disability-associated immobility, if disease activity itself leads to a prothrombotic state.

Extrapolating from this, alternative treatments to IVIG may be a consideration in patients who require high doses of IVIG with predisposing vascular risk factors and immobility. If there is no suitable alternative, then caution with a low threshold for concomitant thromboprophylaxis might be considered.

Distinct from CIDP, autoimmune nodopathies (AN) are immuno-pathologically different forms of inflammatory neuropathy. Autoimmune nodopathies (AN) are characterised by antibody mediated dysfunction at the nodes of Ranvier and are predominantly associated with IgG4 antibodies to paranodal proteins such as neurofascin, contactin-1 and CASPR1. Patients are frequently older and respond poorly to typical treatments for CIDP.

In particular, patients with anti-contactin-1 and anti-pan-neurofascin antibodies often present with nephrotic syndrome [89]. Patients with nephrotic syndrome experience thrombotic events through a combination of a loss of anticoagulant proteins, increased liver synthesis of pro-coagulant factors and platelet dysfunction [90]. They are at risk of both arterial and venous thrombosis and bleeding, with an increased hazard ratio of 3.11 (95% CI, 2.60–3.73) for arterial thromboembolism, 7.11 (95% CI, 5.49–9.19) for venous thromboembolism, and 4.02 (95% CI, 3.40–4.75) for bleeding in the first year [15]. Guidelines recommend prophylactic anticoagulation in nephrotic patients with hypoalbuminaemia and additional risk factors for thrombosis [91]. This would be a separate group to consider pharmacologic thromboprophylaxis given their co-existing nephrotic syndrome with added thrombotic propensity.

### 4.4. Inflammatory Myopathies

Inflammatory Myopathies are a heterogeneous group of immune-mediated disorders characterised by chronic muscle inflammation and weakness. Distinct subtypes with unique histopathological, immunological and clinical features, as well as differing treatment responses are recognised. Many have extra-muscular manifestations, including dermatological, pulmonary, cardiovascular, gastrointestinal and oncological associations.

Risk of both venous and arterial thrombotic events is recognised in inflammatory myopathies. A meta-analysis on 9045 patients with dermatomyositis (DM)/polymyositis (PM) showed that inflammatory myositis was associated with an increased risk of VTE, with a pooled odds ratio of 4.31 (95% CI, 2.55–7.29) [92]. The risk of VTE is highest in the first year after diagnosis and increases with age [16].

In an analysis of 535,358 inpatients admitted for autoimmune disorders, Zoller et al. found a significantly increased risk of pulmonary embolism during the first year, particularly in polymyositis or dermatomyositis with an incidence ratio of 16.44 (95% CI, 11.57–22.69) [74]. In comparison, the overall risk of pulmonary embolism during the first year after admission for an autoimmune disorder was 6.38 (95% CI 6.19–6.57) [74]. The authors suggested that these diseases be regarded as hypercoagulable disorders [70].

Similarly, Amster et al. found that DM and PM patients had a higher risk of developing PE in a large cohort of over 12,000 DM/PM patients and matched controls [93]. In addition, they found that DM/PM patients were more likely to be seropositive for anti-phospholipid antibodies, and those patients were at an even greater risk of developing PE. It was therefore suggested that DM/PM populations should be screened for anti-phospholipid antibodies and potentially considered for prophylactic treatment for PE.

Increased risk of cardiovascular events was shown in another meta-analysis of 25,433 DM/PM patients, with a relative risk of 2.37 (95% CI, 1.86–3.02) for cardiovascular events in patients with inflammatory myopathies [17]. Subgroup analysis showed a relative risk of 1.76 for ischaemic heart disease and 1.47 for ischaemic stroke [17]. The increase in risk could be due to a combination of atherosclerosis and thrombogenic potential. However, due to the inherent heterogeneity of inflammatory myositis, the extent to which underlying cancer or how different subgroups like anti-synthetase syndrome and immune-mediated necrotising myopathy contributes to thrombotic risk is less clear.

In addition, the rare complication of thrombotic microangiopathy has also been described to occur in dermatomyositis [94]. It is a life-threatening complication that occurs in other connective tissue diseases like anti-phospholipid syndrome and systemic sclerosis and characterised by microangiopathic haemolytic anaemia (MAHA) and non-immune thrombocytopenia. Clinical features result from the MAHA and thrombocytopenia, causing pallor, jaundice, fatigue, petechiae and bruising. End organ ischaemia ranging from acute kidney injury to neurological complications like strokes and seizures occurs due to endothelial damage and vascular occlusion [95]. Early recognition and expedient treatment are essential.

While the risk of thrombosis is well-established in inflammatory myositis, there is no consensus with regard to prophylactic strategies. Myositis guidelines [96,97] do not provide generalisable recommendations for prophylaxis of VTE or arterial thrombosis, in view of limited trial evidence and a heterogeneous population.

Of note, there is a rare but significant risk of spontaneous intramuscular haemorrhage, noted in one retrospective cohort to occur at a incidence rate of 2.38 per 1000 patient years (95% CI, 0.06–12.56), and is associated with heparin use with odds ratio of 4.42 (95% CI, 2.86–7.24) [98]. Therefore, an individualised approach, guided by general medical guidelines, is essential to assess thrombotic and bleeding risk and to inform decisions regarding prophylaxis in this group of myositis patients.

Based on the above data, a thromboprophylactic strategy to consider is to have a lower threshold to administer pharmacologic thromboprophylaxis if the patient with inflammatory myopathy has additional thrombotic risk factors of anti-phospholipid antibody positivity, immobility, underlying malignancy, or poorly controlled disease early in disease onset.

### 4.5. Treatments Used in Inflammatory Neuromuscular Diseases

IVIG is frequently used in the treatment of MG, GBS, CIDP and idiopathic inflammatory myopathies (IIM), with the exception of inclusion body myositis. The association of thrombotic events with the use of IVIG has been reported, but incidences vary widely between patient populations. Proposed mechanisms include increase in plasma viscosity, endothelial injury, vasospasm, and haemodynamic changes during infusion.

In 2013, The US Food and Drug Administration issued a black box warning for thrombosis related to IVIG. However, a meta-analysis of 31 RCTs with 3318 IVIG treated patients and 1811 controls did not find a significant increase in risk of thromboembolic events [99], suggesting that the absolute risk of IVIG for thromboembolic events is low. The authors acknowledged that most of their studied patients were younger, with a fairly low baseline risk of arterial or venous thromboembolic events. Results may therefore not be generalisable to older, higher-risk, or more-immobile patients, especially as most of the observed thromboembolic events occurred in trials involving older patients with these higher-risk characteristics.

A randomised, double-blind, placebo-controlled trial on the efficacy of a second IVIG dose in patients with severe GBS showed increased adverse events, including both arterial and venous thromboembolic events, in patients given the second dose of IVIG [100]. This was even though known pre-existing vascular risk factors were an exclusion criterion for randomisation in the trial. Based on retrospective data from patients with neuroimmunological diseases [14,101], it is reasonable to consider that IVIG may add to the baseline risk of thrombosis due to immobility and systemic inflammation. Currently, there are no evidenced based recommendations to mitigate this risk, although practices of limiting dosage and infusion rates are commonly utilised.

In our clinical practice, we would optimise hydration, slow down IVIG infusion rate and limit dosage, where possible, in patients with pre-existing vascular risk factors and/or with additional thrombotic risk factors of immobility or critical illness. There is still insufficient evidence to recommend routine use of thromboprophylaxis with IVIG use, though this can be discussed with patients who may have had thromboembolic events whilst on IVIG, but have no suitable alternative treatments to IVIG.

Glucocorticoids are also frequently used in the treatment of neuroimmunological diseases. A Danish case-control study [102] studying 38,765 VTE cases with 387,650 matched population controls found that glucocorticoids are associated with an increased risk of VTE, particularly with systemic preparations. The risk was highest in new users, with a risk ratio of 3.06 (95% CI, 2.77–3.38). Risk appears to decrease after discontinuation and showed a dose–response relationship, supporting a biological effect rather than as a result of confounding [102].

The risk of first VTE during periods of exposure with oral glucocorticoids was also evaluated by a self-controlled case series method which examined the risk of recurrent VTE in a cohort design on 2547 patients with VTE [103]. The incidence rate ratio (IRR) of first VTE was 2.53 (95% CI, 1.10–5.72) in the week before starting treatment, 5.28 (95% CI, 2.89–9.53) in the first 7 days of treatment, remained elevated afterwards and decreased to 1.55 (95% CI, 0.85–3.12) after 6 months, as compared to unexposed periods. The hazard ratio for recurrence was 2.72 (95% CI, 1.64–4.78) in treatment periods as compared with no treatment. The authors postulated that the VTE risk is contributed to by both underlying disease and glucocorticoid use [103].

Again, there is insufficient evidence to recommend routine thromboprophylaxis with glucocorticoid use, but the above data suggests the need for vigilance and caution in the administration of immune therapy.

## 5. Neuromuscular Disease Associated with Haematological Malignancies

The intersection between neuromuscular medicine and haematological diseases represents a unique diagnostic landscape, where neurological symptoms frequently herald the presence of an underlying blood dyscrasia. Conditions such as POEMS syndrome and other paraprotein-related diseases (e.g., Waldenstrom Macroglobulinemia/Monoclonal Gammopathy of Unknown Significance (MGUS)/Light Chain (AL) amyloidosis) often enter the differential diagnosis of CIDP, particularly when patients do not respond to standard therapy or present with atypical features including pain, autonomic dysfunction, atypical electrophysiology or systemic features [104]. In these conditions, the underlying haematological disorder also contributes significantly to the overall risk assessment for thrombotic events.

Each of the paraproteinemic neuropathies have distinct neurological phenotypes, representing a heterogeneous group of disorders. The distal acquired demyelinating symmetric neuropathy (DADS) phenotype is associated with an IgM anti-MAG antibody. Painful, multifocal axonal neuropathies can occur with cryoglobulinemias. Prominent autonomic and small-fibre involvement is seen in AL amyloidosis, commonly co-existing with multiple myeloma. A CIDP-like demyelinating or length-dependent sensorimotor axonal peripheral neuropathies can occur with many paraproteinemic diseases, including IgG/A MGUS, POEMS syndrome and Waldenstrom macroglobulinaemia [105].

The overall risk of myocardial infarction, stroke, VTE and bleeding is increased in patients with haematological malignancies. A Danish population-based cohort study found that approximately 20% of patients with haematological malignancies experience a thrombotic or bleeding event necessitating hospital contact in the 10 years after diagnosis [106]. The mechanisms for increased thrombotic and bleeding risk are multifactorial and likely vary between different malignancies. They include patient factors like advanced age and immobility, disease factors like intrinsic procoagulant properties of tumour cells, increased haematocrit and dysregulated inflammatory and angiogenic pathways, and treatment-related factors like central venous catheter use and thrombogenic potential of various drugs [106,107].

### 5.1. POEMS Syndrome

POEMS syndrome is defined by the major diagnostic criteria of polyradiculoneuropathy, monoclonal plasma cell disorder, sclerotic bone lesions, elevated vascular endothelial growth factor and the presence of Castleman’s disease. Minor criteria include organomegaly, extravascular volume overload, endocrinopathy, skin changes, papilloedema, and thrombocytosis or polycythaemia. Features that distinguish the polyneuropathy due to POEMS from that of CIDP include severe leg pain, muscle atrophy and distal predominant weakness [108]. Treatment options include corticosteroids, alkylating agents, immunomodulatory imide drugs (IMiDs), and proteasome inhibitors [109].

Arterial and venous thromboses are known to occur at an increased rate in POEMS syndrome. A retrospective review of the University College London Hospitals (UCLH) POEMS Registry revealed that 30% percent of the included 83 POEMS patients experienced arterial and venous thromboses, with more arterial than venous events. The study included detailed characterisation of both arterial and venous events, in particular the occurrence in relation to treatment and disease activity and VEGF levels. The period of highest risk appeared to be during active disease, before treatment began, and with higher baseline serum vascular endothelial growth factor (VEGF), underscoring the relation to disease activity. It was also noted that thrombotic risk was not immediately ameliorated by treatment, and risk reduction took time. No arterial events occurred with a baseline VEGF < 1000 pg/mL [18].

As such, the primary thromboprophylactic regimen implemented at UCLH includes the use of both prophylactic low molecular weight heparin (LMWH) and a low-dose antiplatelet agent after presentation and during treatment, which is continued until the serum VEGF falls below 1000 pg/mL, at which point the antiplatelet agent is discontinued unless there are ongoing risk factors. Prophylactic LMWH is then continued while the patient is on maintenance treatment with prothrombotic agents such as lenalidomide. When in remission, and no longer on chemotherapy with suppressed VEGF levels, all thromboprophylaxis is stopped regardless of ambulatory ability [18].

Similarly, a retrospective cohort study of 230 POEMS patients evaluated at the Mayo Clinic, Rochester [19], found that 27% of patients developed thrombosis, with slightly more frequent arterial versus venous events. Stroke accounted for 26% of all thromboses with most events occurring prior to therapy. Association for thrombosis identified in the study included extravascular volume overload, splenomegaly, elevated prolactin, thrombocytosis, elevated haemoglobin and haematocrit. The authors recommended that a minimum of antiplatelet prophylaxis should be considered for all patients at diagnosis given the high overall rates of thrombosis, with the consideration of full anticoagulation being weighed against fall and bleeding risk for patients with elevated thrombotic risk factors identified above, and/or a prior history of thrombosis, especially if they have not yet received POEMS therapy [19,109].

A previous case report also recommended baseline head magnetic resonance angiography in patients with POEMS syndrome prior to starting lenalidomide and dexamethasone due to lenalidomide-induced ischemic cerebrovascular disease [110].

First-line treatment options for POEMS syndrome are also independently associated with thrombotic potential, in particular alkylating agents like cyclophosphamide [111] and the immunomodulatory drugs (IMiDs), thalidomide and lenalidomide [112]. VTE prophylaxis recommendations follow established guidelines in cancer patients [113,114,115]. Of note, specific guidelines of risk-stratified VTE prophylaxis for patients with multiple myeloma on IMiDs [116] do not similarly exist for POEMS patients, despite the use of similar treatment regimens and additional immobility from polyneuropathy.

Overall, based on the current best available retrospective evidence and prevailing clinical practice, increased thrombotic risk in POEMS syndrome should be mitigated by institution of baseline antiplatelet therapy upon diagnosis, with consideration for anticoagulation for select patients with increased thrombotic risk factors, active disease (as guided by VEGF levels) and IMiD therapy (based on multiple myeloma guidelines). Trials are needed to assess the effectiveness of similar approaches.

### 5.2. Waldenstrom Macroglobulinemia (WM)

Waldenstrom Macroglobulinemia (WM) is an indolent lymphoplasmacytic lymphoma (LPL) associated with the production of monoclonal IgM paraprotein. The effects of circulating IgM accounts for many of the clinical manifestations of WM, including peripheral neuropathy, occurring in up to 47% of patients [117], and other central manifestations of hyperviscosity, such as headaches, dizziness and visual problems. The peripheral neuropathy phenotype is most commonly that of a chronic, progressive, sensory-predominant polyneuropathy, although less common manifestations like mononeuropathy multiplex and autonomic involvement may occur [118].

Prothrombotic mechanisms include high IgM levels causing hyperviscosity that impairs microvascular blood flow, promotion of thrombosis through interactions of the paraprotein with blood cells, coagulation factors, and adhesion molecules, tumour cell–related inflammation and procoagulant activity, and elevated procoagulant factors [20]. The IgM paraproteinemia seen in WM is associated with an increased bleeding risk primarily due to the associated hyperviscosity syndrome, causing impaired platelet and coagulation function [119].

A large Swedish population study has shown an increase in risk in venous thrombosis in patients with Waldenstrom Macroglobulinemia/lymphoplasmacytic lymphoma. The authors identified 2190 WM/LPL patients and assessed the occurrence of venous and arterial thrombosis through registry data from 1987 to 2005, comparing them with matched controls. The study showed a hazard ratio of 4.0 (95% CI, 2.5–6.4) in the first year, decreasing to 2.3 (95% CI, 1.7–3.0) at 5 years, and to 2.0 (95% CI, 1.6–2.5) at 10 years for development of venous thrombosis [20]. No increased risk of arterial thrombosis was found. This study provides important retrospective evidence for the significant and sustained increased risk of venous thrombosis in WM/LPL, particularly early after diagnosis, highlighting the need for studies to clarify mechanisms for thrombosis and to evaluate thromboprophylaxis strategies.

Thromboprophylaxis in WM may be considered in the first year after diagnosis and during treatment with thrombogenic treatments. Extrapolating from guidelines for multiple myeloma and cancer-associated venous thrombosis [116], prophylactic anticoagulation may be considered in patients with WM treated with IMiDs [20].

### 5.3. Primary Light Chain (AL) Amyloidosis

Primary light chain (AL) amyloidosis is a multisystem disorder characterised by the deposition of immunoglobulin light chains produced by the underlying plasma cell dyscrasia. Apart from renal and cardiac manifestations, peripheral nerve involvement characteristically presents as a painful length-dependent polyneuropathy, with significant small-fibre and autonomic dysfunction [120].

Both thrombosis and bleeding risks are associated with AL amyloidosis. This was demonstrated in a Greek retrospective study of 450 patients, where VTEs, arterial events and significant bleeding were reported in 6%, 5% and 9% of patients, respectively [21]. Associated risk factors were prior thrombosis, lower albumin, renal dysfunction, higher bone-marrow infiltration, soft-tissue involvement and IMiD therapy [21]. Importantly, the significant haemorrhagic risk had an associated mortality of 19%. Risk of thrombosis and bleeding is highest in the first 6 months after diagnosis, but continues at a lower constant rate through subsequent periods. Further investigation is therefore required to further assess algorithms to determine both clinical bleeding and thrombotic risks, coupled with laboratory evaluation of clotting parameters, in order to make clinical decisions on thromboprophylaxis [21].

Cardiac involvement is frequent in amyloidosis, occurring in 50–80% of patients. Cardiac factors related to thrombosis include heart failure, atrial fibrillation (AF) and atrial myopathy [121]. Atrial fibrillation occurs in 25–75% of patients, and cardiac thrombi are found in up to 30% [122]. This predisposes to embolic strokes, which can be the initial manifestation in some patients [123]. Findings of AF or cardiac thrombi warrant anticoagulation if diagnosed and must be balanced against the concurrent increased risk of bleeding in the setting of gastrointestinal amyloidosis or cytopenias.

### 5.4. Monoclonal Gammopathy of Unknown Significance (MGUS)

MGUS, traditionally considered to be a benign or pre-malignant condition, is increasingly recognised to have implications on the risk of thrombosis. Testing for the abnormal presence of monoclonal proteins is frequently part of the workup for peripheral neuropathies, often leading to the diagnosis of MGUS. Neurologically, patients with MGUS may manifest with symmetric, distal, and sensory-predominant symptoms, mimicking CIDP [105].

Population based studies have found the rates of arterial and venous thrombosis to be raised in MGUS [124]. For example, a large Swedish retrospective study [23] of 5326 MGUS patients compared with matched controls, using population-based data, showed hazard ratios of 3.4 (95% CI, 2.5–4.6) for VTE and 1.7 (95% CI, 1.5–1.9) for arterial events in the first year after MGUS diagnosis, which decreased slightly but remained elevated in the subsequent years [23]. Interestingly, this risk increase was present in IgG/A MGUS but not IgM. This study provides evidence for the association between MGUS and thrombosis, but more research into the underlying mechanisms and thromboprophylaxis strategies is needed.

More recently, a prospective study of over 75,000 individuals screened for MGUS in Iceland identified 3668 patients with MGUS and followed up for a median of 3.7 years. Unlike earlier registry-based studies, MGUS patients were identified from screening, independent of clinical presentation, minimising confounding from other co-morbidities. The authors found that MGUS is associated with a 1.4-fold (95% CI, 1.19–1.73) increased risk of venous thrombosis but not with arterial thrombosis, unrelated to the M protein concentration, suggesting that there may be a subpopulation of MGUS patients with a prothrombotic phenotype independent of the underlying malignant process [22].

Mechanistically, biomarker studies have shown hypercoagulability, endothelial activation and platelet activation, possibly explaining the observed increase in thrombosis [124]. More research is needed to explore how various biomarkers can be used to identify a subpopulation of MGUS patients with thrombotic significance.

While current evidence does not support routine thromboprophylaxis in MGUS, given its modest association with venous thrombosis, it should be considered as an additional risk factor when considering the overall VTE risk in relevant patients.

## 6. Conclusions

Thrombosis is a clinically important yet often underrecognized complication across the spectrum of adult and paediatric neuromuscular disease. Elements of Virchow’s triad—endothelial injury, haemodynamic changes and hypercoagulability—are responsible for thrombosis to varying extents in the spectrum of diseases covered in our discussion. Factors contributing to thrombosis in many neuromuscular diseases include immobility, systemic inflammation, cardiac dysfunction and treatment related factors. In addition, both venous and arterial thrombotic risks vary substantially between different neuromuscular conditions, reflecting distinct pathomechanisms and a heterogeneity of risk factors.

The available evidence to guide disease-specific thromboprophylaxis in neuromuscular populations is currently limited (Table 2). Current practice is guided by general medical, surgical, and critical care and oncological guidelines. While broadly applicable, they may inadequately account for specific risk profiles of selected neuromuscular patients. This includes disease cohorts which have been identified to have a possible elevated risk beyond that of the usual risk expected for their level of immobility and critical illness, such as patients with myotonic dystrophy, idiopathic inflammatory myopathies, and POEMS syndrome.

Furthermore, possible increased bleeding risk may confound decisions around prophylaxis, especially for dystrophinopathies, inflammatory myopathies and neuromuscular conditions related to haematological malignancies. Increased fall risk from restricted mobility in neuromuscular patients also needs to be considered when weighing the benefits of thromboprophylaxis. Non-pharmacologic measures of thromboprophylaxis such as mechanical compression and graduated stockings may also be difficult to institute in patients who still retain limited mobility and who wish to ambulate, or who have contractures related to their neuromuscular condition.

Future priorities should focus on studies of neuromuscular cohorts to evaluate tailored prophylactic strategies and incorporate advances in biomarkers and risk assessment tools. Addressing these unmet needs is essential to move from reactive vigilance towards rational, evidence-based prevention of thrombotic complications in neuromuscular medicine.

## Figures and Tables

**Table 1:** Risks of arterial and venous events.

Condition	Estimated Increased VTE Risk	Estimated Increased Risk of Arterial Events
Amyotrophic Lateral Sclerosis	Increased—22 per 1000 person-years [7] Hazard ratio of 2.7 [8]	Possibly increased: Stroke 7.8 per 1000 person-years [9] Myocardial infarction 26.2 per 1000 person-years [10]
Spinal Muscular Atrophy	Unknown. Events possibly related to increased immobility and surgical procedures.	Risk of stroke related to atrial fibrillation and structural heart abnormalities
Dystrophinopathies	Increased during surgical procedures and hospitalisations	Risk of stroke related to severe cardiomyopathy and atrial fibrillation
Myotonic Dystrophy	Increased—8.3 per 1000 person-years [11]	Risk of stroke related to atrial fibrillation
Myasthenia Gravis	Increased—rate ratio of 2.26 [12]	Not reported
Guillain Barre Syndrome	Increased in GBS patients who received IVIG—odds ratio 1.36 [13]	Not reported
Chronic Inflammatory Demyelinating Polyneuropathy	In patients who received IVIG, 22.3 per 1000 patient-years [14]	In patients who received IVIG, 35.7 per 1000 patient-years [14]
Autoimmune Nodopathies	With nephrotic syndrome, hazard ratio of 7.11 [15]	With nephrotic syndrome, hazard ratio of 3.11 [15]
Inflammatory Myopathies	Increased—Odds ratio of 4.31 [16]	Increased—relative risk of 2.37 [17]
POEMS Syndrome	Up to 30% experienced arterial or venous events, with slightly more arterial events [18,19]	Up to 30% experienced arterial or venous events, with slightly more arterial events [18,19]
Waldenstrom Macroglobulinemia	Increased—hazard ratio of 4.0 in the first year [20]	No increased risk [20]
Light Chain Amyloidosis	6% of patients experienced VTE in a cohort study [21]	5% of patients experienced arterial events in a cohort study [21]
Monoclonal Gammopathy of Unknown Significance	Increased—hazard ratio of 1.4 [22] to 3.4 [23]	Increased—hazard ratio of 1.7 [23] was found in one study, with a more recent study finding no increased risk of arterial thrombosis [22]

**Table 2:** Evidence gaps in thrombosis across spectrum of neuromuscular diseases.

Condition	Epidemiology and Estimates of Risk	Mechanisms of Thrombosis	Prophylactic Strategies
Amyotrophic Lateral Sclerosis	Variable incidence of VTE across studies. Single prospective cohort for arterial thrombosis.	Predominantly immobility driven. Role of inflammation/other mechanisms less studied. Unclear mechanism in arterial thrombosis.	Unclear risk benefit from anticoagulation. Need for disease-specific strategies.
Spinal Muscular Atrophy	Risk has not been systematically studied.	Endothelial dysfunction and vascular autonomic dysfunction.	No prophylaxis data or guideline recommendations.
Dystrophinopathies	Uncertain true thrombotic vs. bleeding risk. Risk is context and disease stage dependent.	Role of dystrophin specific mechanisms poorly defined. General immobility and cardiac dysfunction.	No disease-specific strategies for high-risk encounters, e.g., surgery or advanced immobility.
Myotonic Dystrophy	Best evidence derived from retrospective data.	Hypothesis of toxic effect of RNA causing hypercoagulability requires further study of patients’ coagulation profiles.	No disease-specific strategies for anticoagulation.
Myasthenia Gravis	Evidence derived from administrative datasets.	Predominantly immobility/critical illness driven. Role of inflammation/other mechanisms less studied.	Recommendations extrapolated from critically ill patients. No disease-specific recommendations.
Guillain Barre Syndrome	No studies to identify risk independent of critical illness or IVIG use.	Predominantly immobility/critical illness driven. Role of inflammation/other mechanisms less studied. IVIG use may be contributory.	Recommendations extrapolated from critically ill patients. No disease-specific recommendations.
Chronic Inflammatory Demyelinating Polyneuropathy	Sparse epidemiological data; reliance on extrapolation from IVIG studies.	Immobility and IVIG use may be contributory. Lack of evidence for disease activity related prothrombotic state.	No disease-specific strategies for anticoagulation. Need for systematic study of best practices with IVIG use in patients with thrombotic risk.
Autoimmune Nodopathies.	Risk inferred from nephrotic syndrome; no studies specific for AN.	Likely increased in setting of associated nephrotic syndrome. Unclear if other independent mechanisms contribute.	Guidelines for anticoagulation in nephrotic syndrome.
Inflammatory Myopathies	Multiple retrospective and prospective studies provide strong evidence of arterial and venous thrombosis. Risk for specific subgroups less well-defined.	Heterogenous disease population; unclear if predominant mechanisms for thrombosis are common or subtype specific.	Risk benefit of anticoagulation; considering increase risk of bleeding is not well-established.
POEMS Syndrome	Strong evidence derived from multiple retrospective datasets.	Disease activity-related.	Aspirin use and thromboprophylaxis practice derived from consensus guidance and extrapolated from multiple myeloma guidelines.
Waldenstrom Macroglobulinemia	Retrospective evidence derived from administrative data.	Hyperviscosity related bleeding and thrombotic risk derive from complex mechanisms.	Current practice derived from multiple myeloma guidelines. No disease-specific recommendations for WM.
Light Chain Amyloidosis	Lack of prospective studies.	Multiple complex contributions to thrombotic risk.	No disease-specific recommendations. Strategies needed for competing risk of bleeding and thrombosis.
Monoclonal Gammopathy of Unknown Significance	Strong prospective and retrospective data.	Thrombotic risk not related to M protein concentration. Unclear mechanism for thrombosis in subgroups.	Low absolute risk, thromboprophylaxis not supported by current evidence.

## Data Availability

No new data were created or analyzed in this study.
